# Translocation and protein complex co‐localization of mTOR is associated with postprandial myofibrillar protein synthesis at rest and after endurance exercise

**DOI:** 10.14814/phy2.13628

**Published:** 2018-03-07

**Authors:** Sidney Abou Sawan, Stephan van Vliet, Justin T. Parel, Joseph W. Beals, Michael Mazzulla, Daniel W. D. West, Andrew Philp, Zhong Li, Scott A. Paluska, Nicholas A. Burd, Daniel R. Moore

**Affiliations:** ^1^ Faculty of Kinesiology and Physical Education University of Toronto Toronto Ontario; ^2^ Department of Kinesiology and Community Health University of Illinois Urbana Illinois; ^3^ Division of Nutritional Sciences University of Illinois Urbana Illinois; ^4^ School of Sport, Exercise and Rehabilitation Sciences University of Birmingham Birmingham United Kingdom; ^5^ Roy J. Carver Biotechnology Center University of Illinois Urbana Illinois; ^6^ Department of Family Medicine University of Illinois Urbana Illinois

**Keywords:** Amino acids, immunofluorescence, mRNA translation, mTOR, muscle protein synthesis

## Abstract

Translocation and colocalization of mechanistic target of rapamycin complex 1 (mTORC1) with regulatory proteins represents a critical step in translation initiation of protein synthesis in vitro. However, mechanistic insight into the control of postprandial skeletal muscle protein synthesis rates at rest and after an acute bout of endurance exercise in humans is lacking. In crossover trials, eight endurance‐trained men received primed‐continuous infusions of L‐[*ring*‐^2^H_5_]phenylalanine and consumed a mixed‐macronutrient meal (18 g protein, 60 g carbohydrates, 17 g fat) at rest (REST) and after 60 min of treadmill running at 70% *V*O_2peak_ (EX). Skeletal muscle biopsies were collected to measure changes in phosphorylation and colocalization in the mTORC1‐pathway, in addition to rates of myofibrillar (MyoPS) and mitochondrial (MitoPS) protein synthesis. MyoPS increased (*P < *0.05) above fasted in REST (~2.1‐fold) and EX (~twofold) during the 300 min postprandial period, with no corresponding changes in MitoPS (*P *> 0.05). TSC2/Rheb colocalization decreased below fasted at 60 and 300 min after feeding in REST and EX (*P < *0.01). mTOR colocalization with Rheb increased above fasted at 60 and 300 min after feeding in REST and EX (*P < *0.01), which was consistent with an increased phosphorylation 4E‐BP1^Thr37/46^ and rpS6^ser240/244^ at 60 min. Our data suggest that MyoPS, but not MitoPS, is primarily nutrient responsive in trained young men at rest and after endurance exercise. The postprandial increase in MyoPS is associated with an increase in mTOR/Rheb colocalization and a reciprocal decrease in TSC2/Rheb colocalization and thus likely represent important regulatory events for in vivo skeletal muscle myofibrillar mRNA translation in humans.

## Introduction

Skeletal muscle is constantly remodelled through the synthesis of new proteins and the breakdown of old and/or damaged proteins. To this end, it is well established that muscle protein synthesis is the prime‐regulated variable with nutrient ingestion (Phillips 2009). Therefore, understanding the nutrient sensitivity of muscle protein sub‐fractional synthetic responses (FSR), such as myofibrillar‐ and mitochondrial‐ protein fractions, will advance our understanding of the plasticity of skeletal muscle. For example, myofibrillar protein synthesis rates have been shown to increase in response to amino acid administration at rest (Moore et al. 2009, 2015; Witard et al. 2014) and after endurance (Breen et al. 2011; Rowlands et al. 2015) and resistance exercise (Moore et al. 2009; Witard et al. 2014). There is also evidence that exogenous amino acids may stimulate mitochondrial protein synthesis rates at rest (Bohe et al. 2001, 2003) and after an acute bout of endurance exercise in trained men (Wilkinson et al. 2008). However, these studies applied either a nonphysiological intravenous amino acid infusion (Bohe et al. 2001, 2003) or small repeated feedings (Wilkinson et al. 2008), respectively. It has recently been demonstrated that a relatively large (i.e., 36 g) protein meal stimulates mitochondrial protein synthesis rates in healthy young adults (Beals et al. 2017). It remains to be determined, however, whether the ingestion of a physiologically relevant mixed macronutrient meal with a moderate protein content can stimulate mitochondrial protein synthesis rates at rest. Moreover, it has not been clearly established if endurance exercise and nutrition interact to potentiate the postprandial myofibrillar or mitochondrial protein synthetic response versus resting conditions in healthy adults.

At the molecular level, the mechanistic target of rapamycin complex 1 (mTORC1), an evolutionarily conserved serine/threonine protein kinase, plays a central role in regulating protein synthesis (Bentzinger et al. 2008; Bar‐Peled and Sabatini 2014). To this point, the tuberous sclerosis proteins 1 and 2 (TSC1 and TSC2) have been shown to negatively regulate mTORC1 signaling through their GTPase activity, converting the Ras homolog enriched in brain (Rheb)‐complex into its inactive GDP‐Rheb complex (Inoki et al. 2003; Zhang et al. 2003). This renders Rheb unable to bind and colocalize with mTORC1, which subsequently inhibits the activation of mTORC1 and its downstream targets ribosomal S6 kinase 1 (S6K1) and the eukaryotic initiation factor 4E‐binding protein (4E‐BP1) (Inoki et al. 2003). We have recently demonstrated that resistance exercise‐induced dissociation of TSC2 from Rheb is mirrored by enhanced mTOR‐Rheb association (Song et al. 2017), the latter of which has recently been confirmed to represent mTORC1 rather than mTORC2 complex formation (Hodson et al. 2017a). This result is similar to results in vitro (Garami et al. 2003) and would be consistent with studies that have shown enhanced mTORC1 activity and muscle protein synthesis during recovery from resistance exercise (Moore et al. 2009; Song et al. 2017). Although resistance exercise alone has recently been shown to induce mTOR‐Rheb colocalization and subsarcolemmal translocation (Hodson et al. 2017a), the impact of endurance exercise to mediate these intracellular signalling events in human skeletal muscle is currently unknown. Moreover, it is unclear how mTORC1 protein‐protein interactions and translocation events align with measured fraction‐specific rates of muscle protein synthesis as studies to date have only utilized proxy markers of mRNA translation (i.e., kinase assays) (Hodson et al. 2017a; Song et al. 2017).

Accordingly, the purpose of our study was to characterize the effect of mixed macronutrient ingestion at rest and after endurance exercise on the regulation of novel molecular events and the subsequent modulation of postprandial myofibrillar and mitochondrial protein synthesis rates in vivo in humans. We hypothesized that: (1) myofibrillar and mitochondrial protein synthesis would be upregulated above fasted values in response to mixed meal ingestion at rest; (2) treadmill running at 70% *V*O_2peak_ would further stimulate postprandial myofibrillar and mitochondrial protein synthesis rates versus feeding alone, and; (3) changes in the phosphorylation and colocalization/subcellular localization of the mTORC1 signalling cascade would be consistent with the enhanced postprandial protein synthetic response at rest and after endurance exercise.

## Methods

### Participants and ethical approval

Eight healthy active young (age: 25 ± 2 years, weight: 72.4 ± 2.7 kg, lean body mass: 60.4 ± 2 kg; *V*O_2peak_: 62.5 ± 2.5 mL/kg/min; means ± SEM) males were recruited for this study. The participants were part of a larger investigation being conducted in our laboratories (Mazzulla et al. 2017; Niemiro et al. 2017). All participants were deemed healthy based on responses to a routine medical screening questionnaire and a physical activity readiness questionnaire (PAR‐Q), and had no prior history of participating in stable isotope tracer experiments. Participants were trained runners who engaged in endurance exercise three to six times per week and were recruited from various running clubs at the University of Illinois Urbana‐Champaign and greater community. Participants provided written consent to a protocol that was approved by the University of Toronto Research Ethics Board and the University of Illinois at Urbana‐Champaign Institutional Review Board and conformed to standards for the use of human participants in research as outlined in the seventh revision of the Declaration of Helsinki.

### Experimental design

Participants underwent two pretesting visits. During the first pretesting visit participants underwent body composition assessment via dual‐energy X‐ray absorptiometry (QDR 4500A; Hologic, Bedford, MA). Moreover, *V*O_2peak_ was determined by a graded treadmill exercise test and gas flow analysis. On a subsequent pretesting visit, participants reported to the laboratory to perform a familiarization exercise trial. During this familiarization trial, participants performed 60 min of treadmill running in order to determine the treadmill settings for the experimental exercise trial. Treadmill settings were adjusted during the first 5 min to elicit an intensity of 70% *V*O_2peak_, as measured by indirect calorimetry, while running at 1% grade and maintained for 60 min. After the familiarization trial, participants were randomly assigned so that half the participants participated in REST or EX conditions for their first infusion trial. Participants were also instructed to refrain from any form of vigorous physical exercise for 72 h prior to the infusion trial. The participants were instructed to consume their regular diet for 2 days prior to the start of the first trial day. Participants were also instructed to record their food intake during those days in a 2‐day food dairy. For the subsequent trial day, participants were instructed to consume a similar diet as was consumed before the first trial day, using the 2 day food diary as their guidance. Their recorded diets included a balance of carbohydrate (3.74 ± 0.37 g·kg^−1^), protein (1.66 ± 0.17 g·kg^−1^·day^−1^ g·kg^−1^) and fat (1.35 ± 0.17 g·kg^−1^) and an energy intake of 34.7 ± 3.4 kcal·kg^−1^ day^−1^ (mean ± SEM).

### Infusion protocol

**Figure 1:**
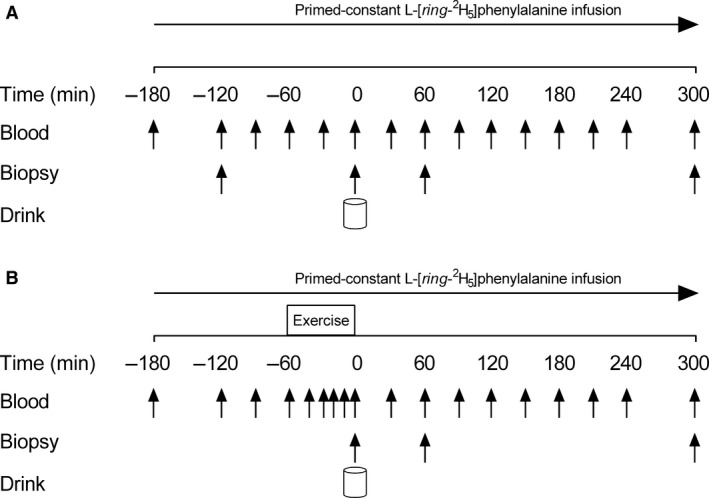
Schematic of the experimental infusion protocol during the rest (REST; (A) and exercise (EX; (B) trials. Trials were randomized and counterbalanced. EX trial involved 60 min of treadmill running at 70% *V*O_2peak_. Vertical arrows indicate blood and muscle biopsy sampling points. The drink was a mixed macronutrient beverage that consisted of 18 g of egg protein +60 g of carbohydrates +17 g of fat.

On trial days, participants reported to the laboratory at 0700 h after an overnight fast. A Teflon catheter was inserted into an antecubital vein for baseline blood sample collection (*t *=* *−180 min). Subsequently, the participants received a priming dose of L‐[*ring*‐^2^H_5_]phenylalanine (2.0 *μ*mol·kg^−1^), prior to initiating a continuous infusion of L‐[*ring*‐^2^H_5_]phenylalanine (0.05 *μ*mol·kg^−1^·min^−1^) using a calibrated infusion pump (PHD 2000, Harvard Apparatus, Natick MA). All infusate was filtered through a sterile filter (0.2 *μ*m). A second Teflon catheter was inserted in a dorsal hand vein and placed in a heated blanket (60°C) for repeated arterialized blood sampling and remained patent with a 0.9% saline drip. During the REST trial, muscle biopsies were collected at *t *=* *−120 and 0 min of infusion to determine fasted rates of muscle protein synthesis (Fig. 1A) and muscle signaling via immunofluorescence and western blotting (for *t* = 0 min only). During the EX trial, only one fasted muscle biopsy was collected at *t* = 0 min (Fig.  1B) for fasted protein‐bound tracer enrichment and muscle signaling via immunofluorescence and western blotting. This 0 min biopsy during EX condition was collected immediately after 60 min of treadmill running at 69.3 ± 0.01% *V*O_2peak_, while participants remained rested on an infusion bed during REST. In both trials, the 0 min biopsy was immediately followed by the ingestion of a mixed macronutrient meal consisting of 18 g of egg protein, 60 g of carbohydrates (50% dextrose and 50% maltodextrin), and 17 g of fat. Postprandial muscle protein synthesis rates were determined between 0 and 300 min. On both trial days, we also obtained a muscle biopsy at 60 min after meal ingestion to determine muscle signaling via immunofluorescence and western blotting. We opted to obtain this muscle biopsy at 60 min to capture peak mTORC1 activation in response to feeding and/or exercise (Camera et al. 2010; Moore et al. 2011; West et al. 2011). All biopsies were collected from the middle region of the *vastus lateralis* (15 cm above the patella) with a Bergström needle modified for suction under local anesthesia (Bergstrom 1975). Fasted muscle biopsies were collected from the same incision with the needle pointed to the distal and proximal directions, respectively. Muscle biopsies obtained at *t = *60 and 300 min were collected from the same leg through separate incisions (2–3 cm apart). During the second trial, the contralateral leg was biopsied, with the order of legs being randomized. All muscle biopsy samples were freed from any visible blood, adipose, and connective tissue, immediately frozen in liquid nitrogen, and stored at −80°C until subsequent analysis. During both trials (REST and EX), blood draws were obtained before (*t *= −180, −120, −90, −60, −30, and 0 min) and after (*t *=* *30, 60, 90, 120, 150, 180, 210, 240, and 300 min) feeding to determine plasma insulin concentrations and L‐[*ring*‐^2^H_5_]phenylalanine enrichment. During the EX trial, additional blood draws occurred while participants were running (*t *= −40, −20, and −5) to confirm isotopic steady state. Blood samples were immediately analyzed for whole blood glucose and lactate concentrations (2300 Stat Plus; YSI Life Sciences, Springs, OH) and subsequently centrifuged at 3000*g* for 10 min at 4°C. Plasma samples were subsequently stored at −20°C for future analysis.

### Plasma analysis

Plasma L‐[*ring*‐^2^H_5_]phenylalanine enrichments were determined by GC‐MS (Agilent 7890A GC/5975C; MSD) as previously described (Burd et al. 2010, 2015). Plasma insulin concentrations were determined by solid two‐site enzyme immunoassay (Mercodia Diagnostics, Uppsala, Sweden), according to the manufacturers protocol. Plasma leucine concentration was determined by liquid chromatography tandem mass spectrometry (1290 HPLC; Agilent Technologies, Santa Clara, CA; 5500 Q‐Trap MS; Sciex, Framingham, MA) as previously described (Dietzen et al. 2008).

### Muscle analyses

Myofibrillar and mitochondrial protein bound enriched fractions were extracted from ~100 mg of wet muscle using the differential centrifugation method as described in detail previously (Burd et al. 2015). Briefly, muscle was homogenized using a glass homogenizer in ice‐cold homogenization buffer (10 *μ*L/mg; 0.01 mol/L sucrose, 0.01 mol/L Tris·HCl, 0.05 mol/L KCl, 0.001 mol/L EDTA) supplemented with protease and phosphatase inhibitor cocktail tablets (Complete Mini, PhosSTOP; Roche Applied Science, Mannheim, Germany). Myofibrillar and mitochondrial protein enriched protein fractions were hydrolyzed by adding 6 mol/L HCl and heated at 110°C for 24 h. The free amino acids were purified using cation exchange chromatography (Dowex 50W‐X8‐200 resin; Acros Organics, Geel, Belgium) before analysis by 5500 QTRAP LC/MS/MS (Sciex, Framingham, MA) as described previously (van Vliet et al. 2016). The L‐[*ring*‐^2^H_5_]phenylalanine enrichments of the mitochondrial‐ and myofibrillar protein‐bound samples were determined by multiple reaction monitoring (MRM) at *m/z* 166.0 → 103.0 and 171.0 → 106.0 for unlabeled and labeled L‐[*ring*‐^2^H_5_]phenylalanine, respectively. Software Analyst 1.6.2 was used for data acquisition and analysis.

### Muscle protein synthesis calculations

FSR of myofibrillar and mitochondrial proteins were calculated using the standard precursor‐product equation with the weighted mean plasma L‐[*ring*‐^2^H_5_]‐phenylalanine enrichment as an estimate of precursor pool enrichment.

### Immunofluorescence

Muscle biopsy samples (~25 mg) were mounted in Optimal Cutting Temperature Compound (Tissue‐Tek^®^, Sakura Finetek USA Inc., Torrence, CA) and frozen in isopentane cooled by liquid nitrogen prior to storage at −80°C for subsequent immunofluorescence analysis. Serial cross‐sections (7 *μ*m) were collected from embedded muscle samples onto room temperature uncoated glass slides. Sections were stained for mTOR colocalization as previously described (Song et al. 2017).

Slides were observed under an EVOS FL Auto Cell imaging microscope (Thermo Fisher Scientific, Waltham, MA) at 40× 0.75NA magnification. For image capture, a DAPI UV (340–380 nm) filter was used to view WGA‐350 (blue) signals, and mTOR stains tagged with Alexa 594 fluorophore (red) were visualized under the Texas red (540–580 nm). GFP (465–495 nm) excitation filter was used to capture signals of mTOR‐associated proteins, which were conjugated with Alexa Fluor 488 fluorophore. All image capturing parameters were kept constant between images, including exposure time, gain, image frame, and light intensity. For colocalization analysis, at least six images were captured per section with each image including ~6 fibres on average. For each subject, muscle samples were taken from three time points/trial (six total), and averaged values were calculated from each time point. As such, the total fibre numbers analyzed for each subject was at least ~275 fibres, which were randomly selected and imaged under the same capture settings. To assess the non‐specificity/autofluoresence of our antibodies, primary antibody was omitted from a single cross section on each slide. Fluorescence intensity of these secondary‐only sections was subsequently utilized as a set point for determining positive staining on all other sections on the slide. All image processing and automated quantitation was carried out in ImagePro Premier v 9.2 (Media Cybernetics Inc., Rockville, MD) where Pearson's correlation coefficient was used to quantify correlations between mTOR/TSC2 with Rheb and WGA. To confirm the suitability of our microscope for our automated analysis we compared it to an inverted confocal microscope (Leica DMI8 connected to an Andor Diskovery Multimodal Imaging System, using an Andor Zyla 4.2 Megapixel sCMOS camera under 40 × 1.30 NA oil immersion objective). We randomly selected *n *= 3 participants and performed mTOR/Rheb and TSC2/Rheb costains prior to image capture using the following filters: (1) DAPI UV (359–461 nm) for WGA‐350 (blue) signals; (2) Alexa 594 fluorophore (red) were visualized under RFP (596–620 nm) for mTOR/TSC2, and; (3) GFP (489–508 nm) for Rheb signals, which were conjugated with Alexa Fluor 488 fluorophore. All image capturing parameters were kept constant between images, including exposure time, gain, image frame and light intensity. There was strong agreement between our widefield microscope and an inverted confocal for mTOR/Rheb (*r* ^2^ = 0.67, *P *<* *0.001) and TSC2/Rheb (*r* ^2^ = 0.87, *P *<* *0.001) costains (data not shown).

### Western blotting

Protein concentrations of the extracts were determined using the BCA assay (Thermo Fisher Scientific, Rockford, IL). Samples were prepared to equal concentrations by dilution with the homogenization buffer used to extract the myofbrillar and mitochondrial protein enriched fractions. Lysates were denatured with Laemmli sample buffer and heat (95°C for 5 min). Samples (10 *μ*g) were loaded on 4–20% gradient polyacrylamide gels and were separated by electrophoresis (200 V for 45 min), and subsequently transferred to nitrocellulose membrane (wet transfer, 100 V for 60 min). Membranes were blocked at room temperature for 60 min using 5% (wt/vol) skim milk in Tris‐buffered saline with 0.1% Tween 20 (TBST) before overnight incubation in primary antibody at 4°C. Primary antibodies, diluted 1:1000 in TBST and 3% milk, were from Cell Signaling Technologies (Danvers, MA) as follows: phospho‐S6K1^Thr389^ (cat. 9205), phospho‐eEF2^Thr56^ (cat. 2331), phospho‐4E‐BP1^Thr37/46^ (cat. 9459), phospho‐mTOR^Ser2448^ (cat. 2971), phospho‐p38 MAPK^Thr180/Tyr182^,phospho‐ERK^Thr202/Tyr204^ (cat. 4377), and phopsho‐ rpS6^ser240/244^ (cat. D68F8; in 5% BSA). Following overnight incubation, membranes were washed in TBST (3 × 5 min) and incubated in secondary antibody (1:20,000 in 3% milk) for 60 min at room temperature before washing in TBST (3 × 5 min) and detecting by chemiluminescence (Millipore; cat. WBKLS0500). Membranes were imaged using a Fluorochem E Imaging system (Protein Simple; Alpha Innotech, Santa Clara, CA). Bands were quantified using Protein Simple AlphaView SA software and normalized to Ponceau S as a loading control (Romero‐Calvo et al. 2010; Rivero‐Gutierrez et al. 2014).

### Statistical analysis

A within‐subject crossover design was used for this study. Muscle protein synthesis and mTOR signaling data were analyzed by two‐way repeated measures ANOVA (time × condition) with Tukey post hoc analysis when *P < *0.05. For colocalization analysis we analyzed the raw Pearson's *r* values for statistical purposes and presented the data as fold‐change to better reflect the physiological changes. Plasma L‐[*ring*‐^2^H_5_]‐phenylalanine enrichment was analyzed using linear regression. GraphPad version 6.00 for Windows (GraphPad Software, San Diego, CA) was used for all statistical analyses. All data are expressed as means ± SEM.

## Results

### Plasma enrichments and metabolites

**Figure 2:**
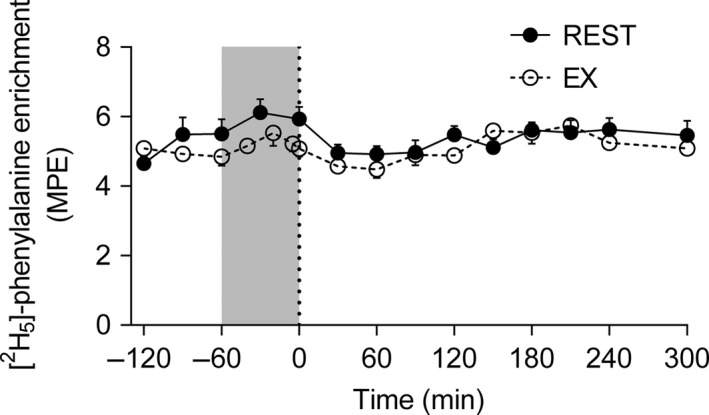
Plasma L‐[ring‐^2^H_5_]phenylalanine enrichment (mole percent excess; MPE) during the REST and EX trials. Circles represent REST, open circles represent EX, gray area represents exercise bout (60 min treadmill running at 70% *V*O_2peak_), and dotted line refers to mixed meal ingestion. Data were analyzed using linear regression. Plasma L‐[ring‐^2^H_5_]phenylalanine: linear regression, *P *=* *0.54; *r* ^2^ = 0.003. Data presented as mean ± SEM.

Plasma L‐[*ring*‐^2^H_5_]‐phenylalanine enrichments were steady across time (*r* ^2^ = 0.003) and did not differ (*P = *0.54) between REST (5.4 ± 0.4 MPE; average over the whole trial) and EX (5.1 ± 0.4 MPE; average over the whole trial) when measured over the entire infusion period (Fig. 2). Plasma insulin and leucine concentrations have been published previously (Mazzulla et al. 2017). Briefly, plasma insulin increased ~18‐fold and ~14‐fold above baseline at 60 min and returned to baseline by 120 min in REST and EX, respectively. There was ~1.3‐fold increase in plasma leucine above baseline at 60 min that returned to baseline by 120 min in both REST and EX.

### Myofibrillar and mitochondrial FSR

**Figure 3:**
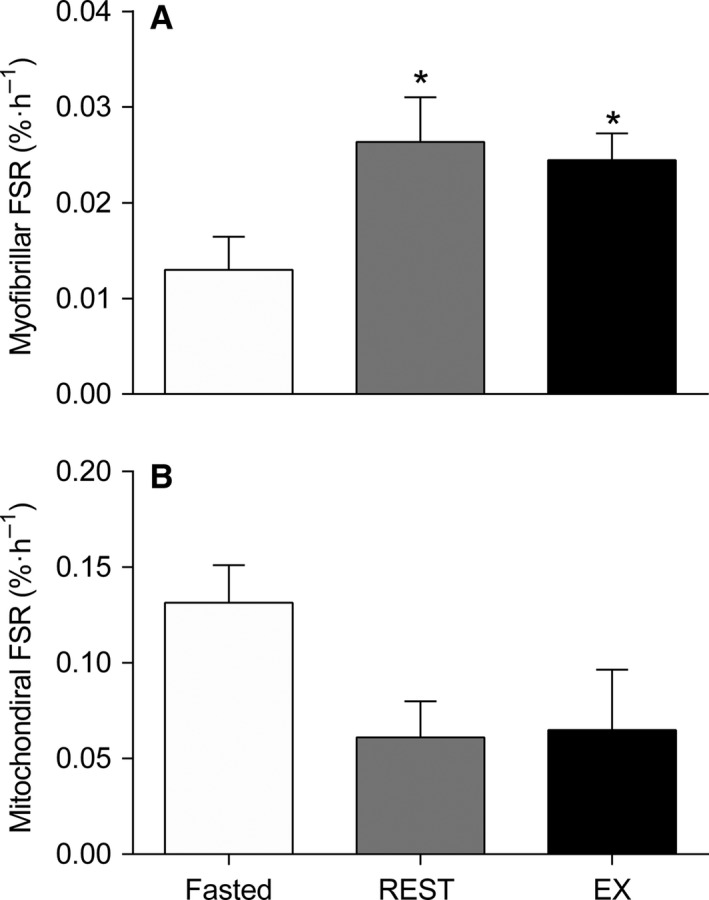
Myofibrillar (A) and mitochondrial (B) fractional synthetic rates (FSR) in the fasted state and after meal ingestion at rest (REST) and after 60 min of treadmill running at 70% of *V*O_2peak_ (EX) throughout a 0–300 min postprandial period in young men (*n *=* *8). Data were analyzed using a two‐way ANOVA. Data presented as mean ± SEM. *Different from Fasted, *P < *0.05.

Myofibrillar and mitochondrial protein‐bound L‐[*ring*‐^2^H_5_]‐phenylalanine enrichments are shown in Table 1. Myofibrillar protein fractional synthesis (FSR) rates were increased (*P < *0.01; Fig. 3A) above fasted values (0.013 ± 0.003) after mixed meal ingestion in the REST (0.026 ± 0.005) and EX conditions (0.024 ± 0.003) during the 300 min postprandial period. Myofibrillar protein FSR was not different (*P = *0.67) between REST and EX conditions. There was no effect of feeding on mitochondrial protein FSR in the REST or EX conditions (*P = *0.14; Fig. 3B).

**Table 1:** Myofibrillar and mitochondrial‐ protein bound L‐[*ring*‐^2^H_5_]phenylalanine enrichments as expressed as mole percent excess (MPE) in response to mixed meal ingestion at rest (REST) and after 60 min of treadmill running at running at 70% of *V*O_2peak_ (EX) in young men (*n *=* *8)

	Time (min)		
Condition	Fasted	0	300
Myofibrillar			
REST (MPE)	0.011 ± 0.003	0.012 ± 0.002	0.019 ± 0.003^a^^,^ b
EX (MPE)		0.010 ± 0.002	0.017 ± 0.003^b^
Mitochondrial			
REST (MPE)	0.022 ± 0.004	0.037 ± 0.005^c^	0.053 ± 0.008^a^^,^ b
EX (MPE)		0.029 ± 0.005	0.045 ± 0.009^b^

Data are mean ± SEM. Data were analyzed using a two‐way repeated‐measures ANOVA.

a Different from Fasted (*P < *0.01).

b Different from 0 min (*P < *0.01).

c Different from Fasted (*P < *0.001).

### Immunofluorescence

**Figure 4:**
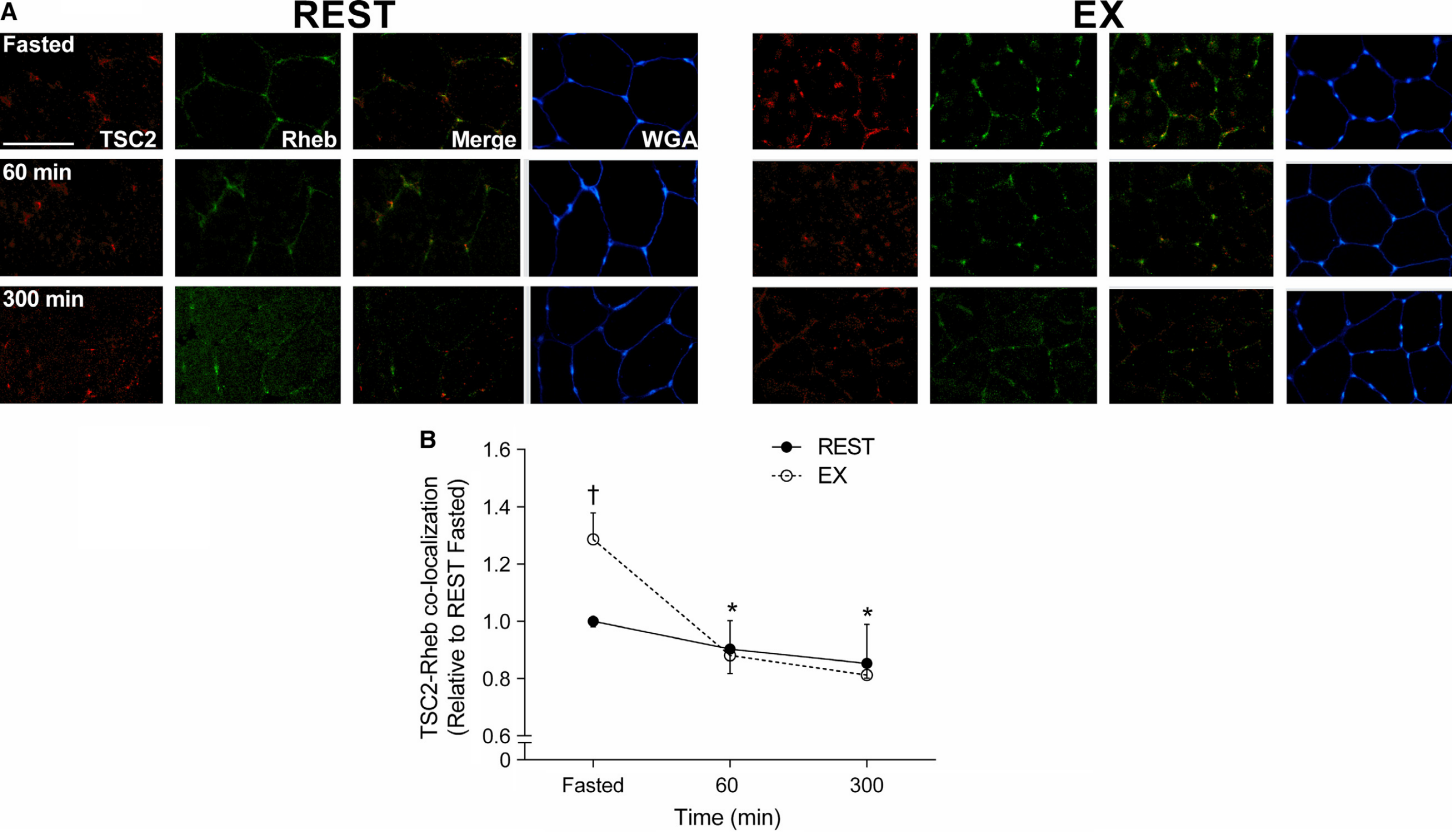
Immunofluorescence quantification of TSC2 (Red) and Rheb (Green) interaction, displayed as a composite image (Merge) and WGA (Blue). Yellow/orange regions represent TSC2 and Rheb interaction. (A) Each panel represents one subject from REST and EX across the experimental time course. (B) Group data are quantified and reported; circles represent REST, open circles represent EX. All data are presented relative to REST Fasted. Scale bar = 100 *μ*m area. Data were analyzed using a two‐way repeated measures ANOVA. Data presented as mean ± SEM. *Different from Fasted in REST and EX (*P < *0.01). ^†^Different from REST at same time point (*P < *0.001).

**Figure 5:**
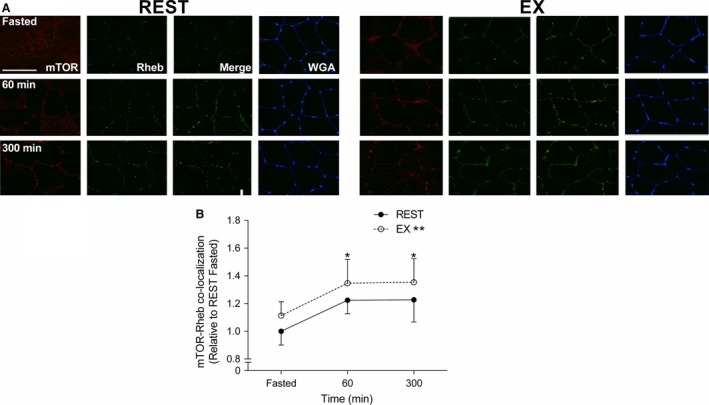
Immunofluorescence quantification of mTOR (Red) and Rheb (Green) interaction, displayed as a composite image (Merge) and WGA (Blue). Yellow/orange regions represent mTOR and Rheb interaction. (A) Each panel represents one subject from REST and EX across the experimental time course (B) Group data are quantified and reported; circles represent REST, open circles represent EX. All data presented relative to REST Fasted. Scale bar = 100 *μ*m area. Data were analyzed using a two‐way repeated measures ANOVA. Data presented as mean ± SEM. *Different from Fasted in REST and EX (*P < *0.01).

**Figure 6:**
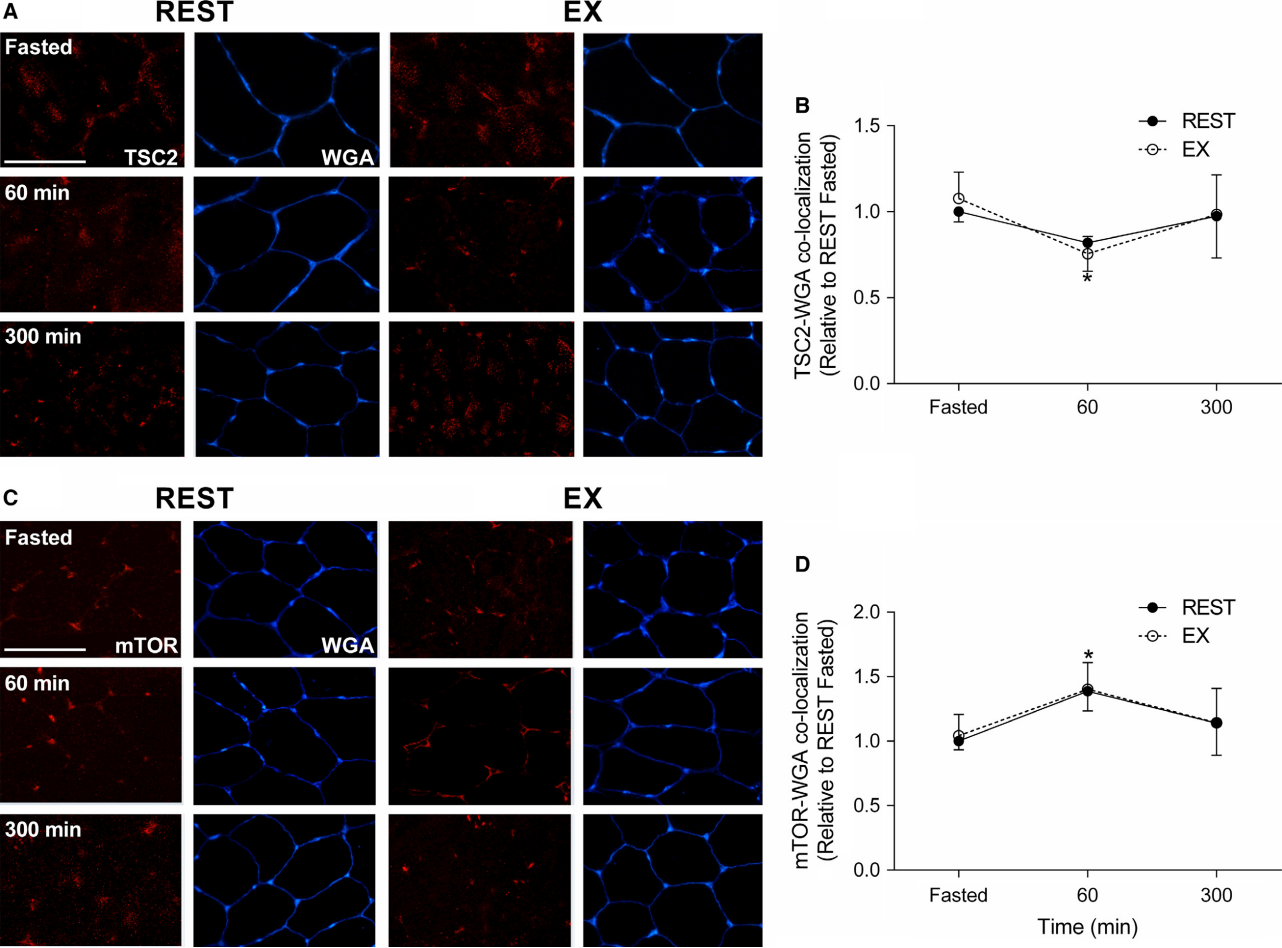
Immunofluorescence quantification of TSC2 (Red) and WGA (Blue) interaction, displayed as individual images. (A) Each panel represents one subject from REST and EX across the experimental time course. (B) Group data are quantified and reported. Immunofluorescence quantification of mTOR (Red) and WGA (Blue) interaction, displayed as individual images (C). Each panel represents one subject from REST and EX across the experimental time course (D). Group data are quantified and reported; circles represent REST, open circles represent EX. All data presented relative to REST Fasted. Scale bar = 100 *μ*m. Data were analyzed using a two‐way repeated measures ANOVA. Data presented as mean ± SEM. *Different from Fasted in REST and EX (*P < *0.01).

Immediately following exercise, TSC2 co‐localization with Rheb was significantly greater compared to the fasted state (*P < *0.001; Fig. 4). Following meal ingestion, TSC2/Rheb co‐localization in REST and EX decreased to a similar level (*P < *0.01) at 60 and 300 min, when compared to the Fasted state (Fig. 4). In response to feeding, mTOR co‐localization with Rheb similarly increased above the Fasted state in REST and EX, respectively (*P < *0.01) (Fig. 5). TSC2 co‐localization with WGA (which delineates the cell periphery) similarly decreased below the Fasted state during REST and EX at 60 min (*P < *0.01) returning back to baseline at 300 min in REST and EX (Fig. 6A). mTOR co‐localization with WGA increased (*P < *0.01) above the Fasted state during REST and EX at 60 min returning back to baseline at 300 min in REST and EX (Fig. 6D). mTOR co‐localization with lysosome‐associated membrane protein 2 (LAMP2) did not change above the Fasted state in REST and EX (*P = *0.81; data not shown).

### Western blotting

**Figure 7:**
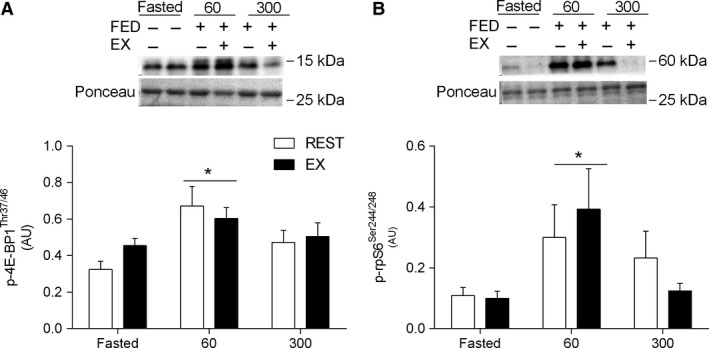
Change in (a) 4E‐BP1^Thr37/46^ and (b) rpS6^ser240/244^ phosphorylation before (Fasted) and after meal ingestion at rest (REST) and after 60 min of treadmill running at 70% of *V*O_2peak_ (EX) in young men (*n *=* *8). Data were analyzed using a two‐way repeated‐measures ANOVA. Data are expressed in arbitrary units (AU). Data presented as mean ± SEM. *Different from Fasted, *P < *0.01.

4E‐BP1^Thr37/46^ phosphorylation was increased above the fasted state (0.33 ± 0.04; *P < *0.01) in REST (0.67 ± 0.1) and EX (0.60 ± 0.06) at 60 min after meal ingestion (Fig. 7A). rpS6^ser240/244 46^ phosphorylation was increased above the fasted state (0.11 ± 0.03; *P < *0.01) in REST (0.31 ± 0.11) and EX (0.4 ± 0.13) at 60 min after meal ingestion (Fig. 7B). There was no effect of exercise or nutrition on other candidate intracellular signaling molecules (i.e., mTOR^ser2448^, S6K1^Thr389^, eEF2^Thr56^). The phosphorylation of ERK1/2^Thr202/Tyr204^ was increased above REST (0.28 ± 0.03; *P = *0.056) at 300 min during EX (0.57 ± 0.06). Furthermore, the phosphorylation of p38 MAPK^Thr180/Tyr180^ was ~1.5‐fold greater during EX than REST (main effect; *P < *0.01; Table 2).

**Table 2:** Change in mTOR^Ser2448^, S6K1^Thr389^, eEF2^Thr56^, p38^Thr180/Tyr180^, Erk1/2^Thr202/Tyr204^ phosphorylation before (Fasted) and after mixed meal ingestion at rest (REST) and after 60 min of treadmill running at 70% of *V*O_2peak_ (EX) in young men (*n *=* *8)

	REST			EX		
	Fasted	60 min	300 min	Fasted	60 min	300 min
p‐mTOR^ser2448^ (AU)	0.37 ± 0.03	0.39 ± 0.04	0.43 ± 0.09	0.35 ± 0.06	0.47 ± 0.1	0.31 ± 0.04
p‐S6K1^Thr389^ (AU)	0.28 ± 0.05	0.41 ± 0.06	0.44 ± 0.07	0.4 ± 0.08	0.31 ± 0.08	0.57 ± 0.12
p‐eEF2^Thr56^ (AU)	0.41 ± 0.03	0.58 ± 0.07	0.57 ± 0.05	0.46 ± 0.06	0.39 ± 0.05	0.51 ± 0.09
p‐p38^Thr180/Tyr180^ a(AU)	0.41 ± 0.06	0.34 ± 0.06	0.34 ± 0.07	0.62 ± 0.09	0.48 ± 0.07	0.55 ± 0.09
p‐Erk1/2^Thr202/Tyr204^ (AU)	0.46 ± 0.17	0.38 ± 0.07	0.28 ± 0.03	0.37 ± 0.09	0.35 ± 0.06	0.57 ± 0.06^b^

Data presented as mean ± SEM. Data were analyzed using a two‐way repeated‐measures ANOVA.

a Main effect of condition; EX>REST *P < *0.001.

b Different from REST at same time point, *P = *0.056.

## Discussion

In this study, we sought to identify the effect of mixed meal ingestion on mTOR signaling events and the subsequent modulation of specific muscle protein sub‐fractional responses with and without prior performance of treadmill running (70% *V*O_2peak_) in endurance‐trained males. We studied a trained population to ensure the exercise‐induced stimulation of muscle protein synthesis was a refined protein fraction‐specific synthetic response rather than the more global damage response that is characteristic of untrained individuals performing a novel bout of exercise (Coffey et al. 2006; Wilkinson et al. 2008; Damas et al. 2016). We demonstrate that mixed meal ingestion consisting of a moderate dose of whole egg (~18 g) protein stimulated postprandial myofibrillar protein synthesis rates at rest. These findings are consistent with previous studies that used isolated protein sources (Moore et al. 2009; Witard et al. 2014) and mixed macronutrient beverages (Churchward‐Venne et al. 2014) to stimulate the postprandial myofibrillar protein synthetic response in the resting‐state. However, in contrast to our hypothesis, an acute bout of treadmill running prior to mixed meal ingestion did not further enhance postprandial rates of myofibrillar protein synthesis throughout a 5 h postprandial period. This result may be related to the trained nature of our population and the sub‐maximal exercise intensity (Wilkinson et al. 2008), which collectively could have resulted in an insufficient exercise stimulus to enhance myofibrillar protein remodeling. Moreover, our results are in contrast to resistance exercise, which augments postprandial myofibrillar protein synthesis rates regardless of training status (Wilkinson et al. 2008; Moore et al. 2009; Witard et al. 2014). Thus, the often presumed interactive nature of nutrition and exercise to stimulate muscle protein synthesis rates, regardless of the assessed muscle protein sub‐fraction, seems to be less apparent during recovery from endurance exercise, at least in the immediate recovery period (0–5 h), versus resistance exercise (Biolo et al. 1997; Burd et al. 2011).

In disagreement with our hypothesis, there was no effect of mixed macronutrient meal ingestion on the stimulation of postprandial mitochondrial protein synthesis rates. Our findings are in contrast to previous studies that demonstrated postprandial mitochondrial protein synthesis rates are responsive to non‐physiological anabolic stimuli such as intravenous amino acid administration (Bohe et al. 2001, 2003) and/or supra‐physiological insulin concentrations (Stump et al. 2003). Furthermore, our data are in contrast to recent findings from some of the present authors that showed postprandial mitochondrial protein synthesis rates are elevated above basal values after lean pork ingestion (36 g of protein and 3 g fat) in healthy young men and women (Beals et al. 2017). Whereas it is relatively well‐established that myofibrillar protein synthesis rates plateau after the ingestion of ~20 g of isolated high quality protein (Witard et al. 2014; Moore et al. 2015), it is unclear what dose of protein consumed as a bolus (either with or without macronutrient co‐ingestion) is required to stimulate resting mitochondrial protein synthetic rates. Thus, the incongruence between our findings and that of others (Witard et al. 2014; Moore et al. 2015) may be related in part to the moderate amount of protein provided in this study (18 g of protein), which may have been insufficient to stimulate postprandial mitochondrial protein synthesis rates above the relatively high basal turnover rates in our trained population.

We also observed that the performance of endurance exercise prior to feeding did not enhance postprandial mitochondrial protein synthesis rates in trained males during the immediate post‐exercise period. It is unclear if our exercise modality, intensity, and/or duration was suboptimal to stimulate mitochondrial protein synthesis rates in our trained population, although the increased p38^Thr180/Tyr180^ phosphorylation would be consistent with a stimulus for mitochondrial biogenesis (Wright et al. 2007) that may have occurred beyond the 300 min recovery period measured in this study (Di Donato et al. 2014). Notwithstanding the possibility of suboptimal exercise stimulus, it was somewhat surprising that there was a visual and perhaps statistical (i.e., *P *=* *0.14) trend towards a moderate suppression in postprandial mitochondrial synthetic rates both at rest and after exercise despite a robust stimulation of myofibrillar protein synthesis rates. The normal diurnal catabolism of skeletal muscle in the fasted state is enhanced during exercise (Howarth et al. 2010) and may primarily target myofibrillar proteins (Goll et al. 2008). We recently demonstrated that consumption of 18 g of egg protein was insufficient to replace exercise‐induced oxidative losses (Mazzulla et al. 2017), which would have included those liberated from muscle catabolism. Thus, it is unclear if there is intracellular partitioning of amino acids towards replenishment of the more abundant, nutritionally sensitive, and slowly turning over myofibrillar protein pool at the expense of the smaller, rapidly turning over mitochondrial protein pool after the consumption of a mixed macronutrient meal containing a moderate ~18 g of dietary protein.

The mTOR pathway is generally considered the main regulator of anabolic stimuli in human muscle (Drummond et al. 2009; Dickinson et al. 2011; Morita et al. 2013). Ultimately, mTORC1 activity is regulated via its interaction with Rheb (Bar‐Peled and Sabatini 2014), the latter of which is negatively regulated by TSC2 (Inoki et al. 2003; Zhang et al. 2003). We observed that TSC2/Rheb co‐localization increased above fasted immediately after endurance exercise, which could be consistent with an exercise‐induced decrease in muscle protein synthesis (Rose et al. 2005; Norton and Layman 2006). However, the endurance exercise‐induced suppression of muscle protein synthesis, which may occur via calcium‐induced suppression of mRNA translation elongation independent of mTORC1 activity (Rose et al. 2005), is not consistently observed in humans (Beelen et al. 2011; Hulston et al. 2011). Thus, the biological significance of a greater post‐exercise co‐localization of TSC2/Rheb, which did not influence mTOR/Rheb co‐localization or mTOR‐related anabolic signaling (e.g., 4E‐BP1, rps6) in the present study, is unclear. In contrast, we observed a decrease in TSC2/Rheb co‐localization at 60 and 300 min after feeding at rest and recovery from endurance exercise. This suggest that the disassociation between the negative regulator TSC2 away from the positive regulator Rheb may play a role in increasing postprandial myofibrillar protein synthesis rates after mixed meal ingestion (Betz and Hall 2013; Marcotte et al. 2015). Our data is an agreement with recent work demonstrating a reduction in TSC2/Rheb co‐localization with a reciprocal increase in mTOR/Rheb co‐localization at 60 and 180 min after resistance exercise and nutrient ingestion (Song et al. 2017). Although we observed a transient increase in 4E‐BP1 and rpS6 phosphorylation at 60 min that would be consistent with similarly enhanced postprandial mRNA translation initiation at rest and after exercise (Fujita et al. 2007; Drummond et al. 2009; Dickinson et al. 2011), there was little effect of nutrient and/or exercise on the phosphorylation of other mTOR candidates (i.e., S6K1). However, the dissociation between static‐point measurements of protein phosphorylation and kinase activity (McGlory et al. 2014) and/or more dynamic measurements of muscle protein synthesis rates (Greenhaff et al. 2008) is not uncommon. Nevertheless, the subcellular co‐localization events reported here and in a previous publication (Hodson et al. 2017a; Song et al. 2017) would be consistent with an increased activity of mTOR and could contribute to the postprandial stimulation of myofibrillar protein synthesis at rest and after endurance exercise. In contrast, the dissociation between the mTOR co‐localization and translocation events and mitochondrial protein synthesis would be at odds with studies in vitro (Morita et al. 2013) but consistent with an mTOR‐independent pathway for the regulation of this muscle protein fraction in rodents (Philp et al. 2015). Given that mTOR‐dependent regulation of mitochondrial protein synthesis in vitro is linked to the increased ATP requirement for global mRNA translation (Morita et al. 2013), it is possible that the apparent dissociation in the present study may also be related to the likely abundant mitochondrial mass in our trained population. Thus, more research is required to elucidate the intracellular signaling events regulating acute mitochondrial protein synthesis in human skeletal muscle.

The exact locus of protein synthesis in human skeletal muscle is largely unknown. Myofibrillar proteins represent ~65% of total muscle proteins with their synthesis suggested to occur within subsarcolemmal regions prior to insertion within functioning sarcomeres (Goll et al. 2008). In contrast, mitochondria exist within both subsarcolemmal and intermyofibrillar pools. Our muscle extraction protocol would have captured both of these mitochondrial pools and thus reflected a “mixed” mitochondrial protein synthetic response (Burd et al. 2015). Moreover, mitochondria possess constituent proteins that are encoded by both peripheral myonuclei and within the mitochondrial DNA itself. Indeed, the free amino acid pool that provides substrates for mRNA translation and thus influences the localization of mitochondrial protein synthesis is currently unknown. In rodent skeletal muscle, ribosomes are generally located around the periphery of muscle cells (Horne and Hesketh 1990). The peripheral ribosomal clustering would be consistent with localization of newly translated polypeptides in vivo as determined by immunofluorescence visualization of puromycin in rodent muscle (Goodman et al. 2012), although the nature of these new peptides (e.g., myofibrillar vs. mitochondrial) is unclear. However, the presence of ribosomes in close proximity to the capillaries that deliver anabolic nutrients and amino acid transporters responsible for their uptake (e.g., L‐Type Amino Acid Transporter‐1) (Hodson et al. 2017b) would ostensibly position these regulatory proteins to optimally respond to amino acid‐induced enhancement of mRNA translation (Bar‐Peled and Sabatini 2014). In our hands, we observed TSC2 to be located at the cell periphery (as suggested by WGA‐co‐localization) and co‐localized with Rheb in the rested fasted state, which would be consistent with reduced rates of myofibrillar protein synthesis in the absence of an anabolic stimulus (e.g., amino acids and/or exercise) (Moore et al. 2009; Phillips 2009; West et al. 2011). In response to feeding, we observed translocation of TSC2 away from the cell periphery with a corresponding translocation of mTOR towards the cell periphery at 60 min independent of prior contractile activity, which would be consistent with recent observations at rest (Hodson et al. 2017a). The translocation of mTOR towards the cell periphery could be physiologically relevant as this could put mTOR in close proximity to receive amino acid substrates (Song et al. 2017), which could contribute to enhanced myofibrillar protein synthesis observed in the present study. Importantly, the mTOR subsarcolemmal translocation has recently been shown to be mTORC1 (Hodson et al. 2017a), which would be consistent with the role of this complex in regulating cellular anabolism (Bar‐Peled and Sabatini 2014). Previously, some of the present authors have shown that mTOR translocation to the cell periphery is sustained up to 180 min after resistance exercise (Hodson et al. 2017a; Song et al. 2017), which would be consistent with a sustained myofibrillar protein remodelling that may persist for up to 24 h after this exercise modality (Burd et al. 2011, 2012). Thus, our observation of mTOR translocation away from the subsarcolemmal region by 300 min after mixed meal ingestion is consistent with the duration of the postprandial myofibrillar protein synthetic response after protein ingestion (Moore et al. 2009; Atherton et al. 2010; Churchward‐Venne et al. 2014). WGA binds to membrane‐associated glycoproteins that may be found both within the sarcolemmal as well as extracellular connective tissue (Kostrominova 2011), which may complicate the ability to delineate intra versus extracellular immunofluorescence. However, WGA represents an effective means to delineate skeletal muscle cell periphery (Kostrominova 2011) and represents an approach that is consistent with our previous work demonstrating a mTORC1 translocation towards the cell periphery (Hodson et al. 2017a; Song et al. 2017). Moreover, we previously observed that mTOR does not co‐localize with DAPI (Song et al. 2017), suggesting any mTOR‐WGA co‐localization is unrelated to cellular migration outside of the myofibre. Collectively, we interpret our data to suggest that similar to rodents (Goodman et al. 2012), the cell periphery is a hub for human muscle protein synthesis in vivo and could explain in part the similar nutrient‐driven postprandial myofibrillar protein synthetic response at rest and after endurance exercise in healthy adults. The lack of any apparent coordination between mTOR translocation and mitochondrial protein synthesis could reflect the potentially greater complexity associated with translating mRNA from multiple DNA loci and within divergent subcellular locations (i.e., subsarcolemmal and intermyofibrillar).

In conclusion, we show that ingestion of a mixed‐macronutrient meal (containing 18 g of protein) at rest increased myofibrillar protein synthesis rates but not mitochondrial protein synthesis rates in young men. Treadmill running at a moderate intensity (~70% *V*O_2peak_) and duration (60 min) prior to mixed meal ingestion did not potentiate the stimulation of postprandial myofibrillar and mitochondrial protein synthesis rates in this trained population. These data add to our understanding of how muscle specific protein sub‐fractions are regulated and suggest there is a preferential stimulation of contractile protein synthesis versus mitochondrial protein synthesis, irrespective of exercise‐state, when a moderate amount of protein is consumed within a mixed meal in endurance trained males. At the molecular level, our work suggests that the cell periphery translocation of mTOR and its co‐localization with Rheb may regulate postprandial myofibrillar protein synthesis rates but not mitochondrial protein synthesis rates in healthy young adults. Thus, we provide the initial framework to begin to unravel the complexity of the nutritional and exercise regulation of skeletal muscle mass in trained males.

## Disclosures

The authors declare no financial conflicts of interest.
